# New Appliances and Things Medical

**Published:** 1902-08-16

**Authors:** 


### NEW APPLIANCES AND THINGS MEDICAL.

[We shall be glad to receive at our Office, 28 & 29 Southampton Street, Strand, London, W.C., from the manufacturers, specimens of all new preparations and appliances which may be brought out from time to time.]

### RENAGLANDIN AND UNIVERSAL VAPORISER.

(Oppenheimer, Son and Co., Ltd., 179 Queen Victoria Street, London, E C.)

Renaglandin is a concentrated and aseptic solution of extract of suprarenal gland. As a hæmostatic for controlling hæmorrhage in dental practice, and in cases of hæmophilia, this preparation is of great value. It may also be employed in the treatment of pulmonary hæmorrhage: for this purpose it should be diluted, and administered by means of the "Universal Vaporiser." This is an instrument which nebulises fluids so finely that they are rendered practically vapours, and can thus be continuously inhaled in the hope that they will reach the most remote areas of the respiratory mucous membrane. Vapour enters the lungs in the same manner as air, thus differing markedly from the nebulæ from other varieties of spray, which contain such considerable particles that they immediately become condensed on the mucous membrane of the mouth and pharynx without reaching the lungs at all. Renaglandin has also given good results when used in operations on the eye, ear, nose, throat, and urethra, blanching the tissues, thus rendering the operation practically bloodless; moreover, cocaine anæsthesia is considerably prolonged—two very important and valuable considerations in operations on the special organs.

### KEEN'S MUSTARD. ROBINSON'S PATENT BARLEY. ROBINSON'S PATENT GROATS.

(Keen, Robinson and Co., Ltd., Garlick Hill, London, E.C.)

We have before referred in these columns to the excellence and purity of the above preparations. We can only endorse the views we have before expressed. Robinson's Patent Barley can hardly be improved upon for preparing barley water. This diluent and demulcent drink is not nearly so popular as its merits, from the point of view of hygiene, entitle it to be. It can be made a most delicious summer beverage if prepared according to the receipt supplied with each package, or, with rather a smaller quantity of the Patent Barley than advised by the manufacturers. If this beverage is carefully made, well flavoured with lemon and iced, there is no better non-alcoholic drink.

